# A PK/PD model for the evaluation of clinical rifaximin dosage for the treatment of dairy cow mastitis induced by *Escherichia coli*

**DOI:** 10.1186/s12917-022-03564-2

**Published:** 2023-01-21

**Authors:** Honglei Wang, Chen Chen, Chunshuang Liu, Xiaojie Chen, Jingju Zhang, Yufeng Wang, Mingyue Han, Yiming Liu, Xiubo Li

**Affiliations:** 1 National Feed Drug Reference Laboratories, Feed Research Institute, Chinese Academy of Agricultural Sciences, Beijing, China; 2 Laboratory of Quality & Safety Risk Assessment for Products On Feed-Origin Risk Factor, Ministry of Agriculture and Rural Affairs, Beijing, China

**Keywords:** *Escherichia coli*, Mastitis, Rifaximin, PK/PD model, Monte Carlo simulation

## Abstract

*Escherichia coli* (*E. coli*) is an opportunistic pathogen that can cause clinical mastitis in dairy cows worldwide. Mastitis produces severe symptoms in dairy cows, such as udder inflammation, the production of harmful substances, reduced milk production, and altered milk quality. Intramammary injections of rifaximin have a beneficial effect on dairy cow mastitis, especially for mastitis caused by *E. coli*. However, we do not know whether the currently accepted clinical administration scheme is reasonable. Therefore, the purpose of this experiment was to evaluate the clinical dosing regimen for curing mastitis induced by *E. coli*. In this study, the pharmacokinetics of four single dose groups (50, 100, 200, and 400 µg/gland) were studied in CD-1 lactating mice, and the main pharmacokinetic parameters were obtained by non-compartment and two-compartment model of Phoenix 8.1 software. A total of 5,000 colony-forming units (CFU) of *E. coli* ATCC25922 were injected into the mammary glands of mice under anatomic microscope guidance. After 12 h of growth in vivo, the mouse mastitis model was successfully developed. In pharmacodynamics experiment, 12 different dosing regimens (doses ranged from 25 to 800 µg/gland and two dosing intervals of 12 and 24 h) were used to study the therapeutic potential of rifaximin for mastitis. The PK/PD model was established by integrating pharmacokinetics and pharmacodynamics using the inhibitory sigmoid E_max_ model. The optimal antibacterial effect was 2log_10_CFU/gland reduction of bacterial colony counts in vivo, when the magnitude of AUC_24_/MIC exceeded 57.80 h. A total of 57.80 h of AUC_24_/MIC was defined as a target value in the Monte Carlo simulation. The clinically recommended dosage regimen of 100 mg/gland every 12 h in a day achieved a 91.08% cure rate for the treatment of bovine mastitis caused by *E. coli* infection.

**Supplementary Information:**

The online version contains supplementary material available at 10.1186/s12917-022-03564-2.

## Introduction

Mastitis, a disease of the mammary gland, is caused by a local infection by bacteria, fungus, yeast, or even algae. This disease affects almost all mammals, including humans. Cow mastitis is an inflammation of the mammary gland that causes huge economic losses in the dairy industry due to affected milk quality and yield, along with associated treatment costs [1–3]. The occurrence of mastitis in dairy cows is usually due to environmental mastitis pathogens, as the infection usually occurs during the milking process [4]. *E. coli*, a common Gram-negative bacterium and environmental pathogen, is one of the most common pathogenic bacteria causing mastitis [5]. Antibiotic therapy is a good way for treatment of acute mastitis induced by *E. coli*. Rifaximin, a non-aminoglycoside semisynthetic antibiotic derivative of rifamycin, possesses broad-spectrum antibacterial activity against aerobic and anaerobic Gram-positive and Gram-negative microorganisms [6]. Rifaximin acts by binding to the β-subunit of bacterial deoxyribonucleic acid (DNA)-dependent ribonucleic acid (RNA) polymerase enzyme to inhibit RNA synthesis in susceptible bacteria, thus preventing translocation and stopping transcription [7]. Rifaximin has been shown to be efficacious for the prevention of bovine mastitis alone or in combination with cephacetrile or *Melaleuca armillaris* essential oil [8, 9].

Misuse or abuse of antibiotics in farms has led to the enrichment of antibiotics and antibiotic resistance genes in the environment that can cause drug resistance in humans [10]. Antimicrobial resistance is a global problem that affects all countries [11]. It is currently difficult for pharmaceutical companies to develop new drugs because of the long research cycle and high cost of research and development [12]. Therefore, using advanced treatment and optimizing the existing dose scheme constitute the most effective method to solve the problem of bacterial drug resistance. The pharmacokinetic/pharmacokinetic link model (PK/PD) approach is an effective tool in the rational selection of dosage regimens of antimicrobial agents [13]. In the study of mastitis, researchers found that it is expensive to use cows, and the pathological phenomenon of mouse mastitis is similar to that of dairy cows [14]. Therefore, the mouse mastitis model was favored to study the pathogenesis of cow mastitis and optimize the reasonable dosing schemes using PK/PD model for cow mastitis [15–17].

In this study, the PK/PD model of rifaximin was integrated, and the inhibitory sigmoid E_max_ model was used to descript the surrogate PK/PD index required for different levels of antibacterial activity. Additionally, the PK/PD cutoff values were calculated and the PK/PD profiles in mastitis mouse were extrapolated to bovine mastitis. Finally, the clinical dosing schemes of rifaximin for curing dairy cow mastitis induced by *E. coli* were evaluated by the Monte Carlo simulation.

## Materials and methods

### Animals, reagents, and bacterial strains

Healthy lactating CD-1 mice (Charles River Laboratories, Beijing, China) weighing 30–45 g were maintained under specific pathogen-free environment in this study.

Rifaximin was obtained from Sigma Chemical Company, St. Louis, Missouri, USA. Dimethyl sulfoxide (DMSO) was used to dissolve the rifaximin standard at a concentration of 8,000 µg/ml. The working solution was prepared by diluting the stock solution to the appropriate concentration with DMSO.

Liquid chromatography-grade methanol, acetonitrile, and ammonium formate were obtained from Thermo Fisher Technology, China. The type 1 ultrapure water (18.2 MΩ.cm) was delivered by PALL Cascada Purification System, USA.

In 2017–2019, 45 clinical isolates were separately isolated from milk which was produced by 45 dairy cows with mastitis in Beijing. These isolates were identified by chromogenic medium, microscope, and PCR. The reference strain ATCC25922 was obtained from China Agricultural University.

### Susceptibility tests

The minimum inhibitory concentrations (MICs) of rifaximin against 45 clinical *E. coli* strains were determined by broth microdilution method according to the recommendations of the Clinical and Laboratory Standards Institute [18]. *E. coli* ATCC25922 was used as control bacteria in all parallel experiments. The MIC_50_ and MIC_90_ values were calculated, which translate to inhibiting at least 50% and 90% the bacterial population, respectively.

The in vitro time-killing curve experiments of *E. coli* ATCC 25,922 were performed in two groups with an initial bacterial inoculum of 10^6^ and 10^7^ CFU/mL, respectively. These two groups of *E. coli* were exposed to rifaximin at concentrations of 0.5 × , 1 × , 2 × , 4 × , and 8 × MIC, respectively, and grew in a constant temperature incubator at 37 °C with a shaking at a speed of 200 rpm. The number of bacteria in each group were calculated by gradient dilution coating method. The MH agar plates were cultured in constant temperature incubator at 37 °C for 24 h before counting.

### Pharmacokinetics

The mammary glands of the fourth pair are independent of each other and can be used as two independent samples for analysis. This method has been reported in our previous studies [17]. Four single-dose groups, with doses of 50, 100, 200, and 400 µg/gland, were used in the pharmacokinetics experiments. In all, 120 healthy lactating CD-1 mice were selected and randomly separated into four groups, with 30 mice in each group. The mice were anesthetized by intraperitoneal injection of pentobarbital and were injected with rifaximin using a micro-syringe into the gland under a dissecting microscope. The gland tissue samples were collected at 5 min, 10 min, 15 min, 30 min, 45 min, 1 h, 4 h, 8 h, 10 h, 12 h, and 24 h after administration. At each time point, six mammary gland tissues were collected. All mammary gland samples were processed, and rifaximin concentration in the mammary gland was detected by high-performance liquid chromatography (HPLC).

The processing method of mammary gland samples is similar to the method used in our previously published papers [17]. Briefly, mammary gland samples were homogenized, and 0.5 g of mammary gland samples were transferred into a 10-mL polypropylene centrifuge tube. Three milliliters of acetonitrile were also added into each centrifuge tube to extract rifaximin. The mixture was vortexed for 3 min and the samples were centrifuged at 7,104 × g for 5 min. The supernatant was transferred to another tube, and the remaining residue was re-extracted with 3 ml volume of acetonitrile. The two parts of supernatant were added to a solid phase extraction (SPE) cartridge (Oasis HLB 3 cc 60 mg, Waters Company, USA). Then, the SPE cartridge was eluted with 3 ml acetonitrile. The eluent was evaporated to dryness under a stream of N_2_ at 40 °C. Finally, the residue was reconstituted in 1 ml methanol and filtered through a 0.22-µm filter, and then was analyzed by HPLC.

A C_18_ reverse-phase column (Waters XBridge ShieldRP18 4.6 mm × 250 mm, 5.0 μm) was used to separate different substances. The mobile phase comprised methanol, acetonitrile, and ammonium formate (3.16 g/L, PH = 7.2) (V/V/V = 31.5:31.5:37). The flow rate of the mobile phase was 1.4 mL·min^−1^. The injection volume was 20 μl, and the detection wave length of UV was 276 nm. The limits of detection (LOD) and the limits of quantitation (LOQ) were determined by a known concentrations of rifaximin, whose lowest concentration met the requirement of a signal-to-noise ratio of ≥ 3 and ≥ 10, respectively. The accuracy and precision were evaluated by adding a known concentrations of rifaximin (10, 100, or 200 μg/g) to the blank mammary gland in five replicates over five consecutive days. The recoveries and relative standard deviations (RSD) were obtained to judge the accuracy and precision of the method, respectively. The recovery of rifaximin was calculated by rifaximin recovered from gland tissue dividing by the known concentration of the rifaximin standard. The precision was calculated based on the formula as RSD (%) = [SD/M] × 100%, where SD is the standard deviation and M is the average concentration of replicates.

### Pharmacodynamics

The mouse mastitis model caused by *E. coli* was based on previous reports [16, 17, 19]. In brief, the offspring were removed from lactating mice at 2 h before the experiment. The selected mice were anaesthetized with pentobarbital. Then, a small cut was made at the far part of the fourth pair of mammary glands in the mouse abdomen, and 50 µl of 10^6^ CFU/ml of bacterial fluid was injected into the gland through the nick in the mammary gland duct under a stereomicroscope using a 33G micro-syringe.

The pharmacodynamics experiments were divided into 12 therapeutic regimens covering various doses ranging from 25 to 800 µg/gland, and two dosing intervals of 12 h and 24 h per 24-h experiment cycle. A total of 36 lactating mice were used in the pharmacodynamics experiments with three mice in each dosing group. We selected 3 mice with successfully established mastitis model as the control group, which were not treated with rifaximin. The fourth pair of glands from mastitis mice were injected with 100 µl rifaximin. After 24 h of treatment, the mice were euthanized with CO_2_ and dissected to obtain gland samples. Then, the fourth pair of mammary glands were homogenized with a tissue homogenizer and diluted by 0.9% normal saline appropriately. The tissue diluent was inoculated on MH agar plates to count the bacteria. The antibacterial effect of rifaximin is expressed as the decrease in the number of bacteria.

### PK/PD analysis

The inhibitory effect sigmoid E_max_ model of WinNonlin software (version 8.3; Pharsight, USA) was used to analyze the relationship between the antibacterial effect of different doses of rifaximin in the infected mice and the PK/PD parameters (AUC/MIC, T > MIC, C_max_/MIC). According to the simulation equation, the PK/PD target values for bactericidal effects were obtained. The simulation equation is as follows:

$$E = {E}_{\mathrm{max}} - \frac{\left({E}_{\mathrm{max}} - {E}_{0}\right) \times {C}_{e}^{N}}{{EC}_{50}^{N} + {C}_{e}^{N}}.$$

Here, E is the antibacterial effect of rifaximin, which is the bacterial decrease value (△log10CFU/ gland) in the mastitis mice group after 24 h of treatment; E_max_ is the log10CFU/gland in the drug-free control sample; EC_50_ is the value of PK/PD index of drug when the drug produces 50% the maximum antibacterial effect; C_e_ is the parameters of PK/PD model (including T > MIC, AUC/MIC and C_max_/MIC); N is the Hill coefficient, which describes the steepness of the dose–response curve.

### Monte Carlo simulation

The three kinds of clinical regimens of rifaximin in the treatment of mastitis cows were computed by Monte Carlo simulation in Crystal Ball Professional V7.2.2 (developed by the U.S. Oracle company). The pharmacokinetic parameters AUC of rifaximin in the milk sample were assumed to be logarithmically distributed with a mean value and standard deviation of 340.73 ± 43.968 h·μg/ml according to our previous PK study of rifaximin in dairy cows (The results have not been made public, which will be published in another article.). The MIC obeys the custom distribution according to the distribution probability of MIC in the clinic susceptibility tests. The PK/PD target values were obtained from the mice mastitis model experiment. Monte Carlo simulation was performed for 10,000 sessions.

## Results

### Susceptibility tests

Forty-five clinical isolates of *E. coli* were used in MIC tests, and the MIC values of rifaximin ranged from 4 to 16 µg/ml for the clinical isolates in vitro, which are shown in Table 1. In addition, the prevalence and distribution of MICs for 45 clinical *E. coli* strains, being 4, 8 and 16 µg/ml, were 0.222, 0.689, and 0.089, respectively. The MIC_90_ and MIC_50_ calculated were all 8 µg/ml, against the bacterial population of the 45 isolates of *E. coli.*

**Table 1:** The MICs of rifaximin against 45 clinical isolates of *E. coli*

Name of bacteria	MIC (µg/ml)	Name of bacteria	MIC (µg/ml)	Name of bacteria	MIC (µg/ml)
BJS9C023	4	JBW8C0066	8	BJJ9C003	8
BJS9C052	4	BJN8C132	8	BJS9C055	8
BJS9C030	4	BJN8C152	8	BJS9C039	8
BJS9C040	4	BJN8C177	8	BJS9C050	8
BJS9C041	4	BJN8C169	8	BJS9C042	8
BJS9C023	4	BJH8P043	8	BJA8P016	8
BJS9C032	4	XJ28C097	8	BJA89012	8
BJS9C034	4	BJS9C001	8	BJK8C055	16
BJS9C061	4	BJS9C019	8	BJD8C021	16
BJS9C013	4	BJS9C002	8	TJH8C021	16
BJK8C007	8	BJS9C016	8	BJN8C157	16
TJH8C041	8	BJS9C029	8		
BJD8C024	8	BJS9C038	8		
BJD8C030	8	BJS9C058	8		
JBX8C059	8	BJS9C048	8		
TJH8C030	8	BJS9C009	8		
JBW8C0044	8	BJS9C007	8		

**Fig. 1:**
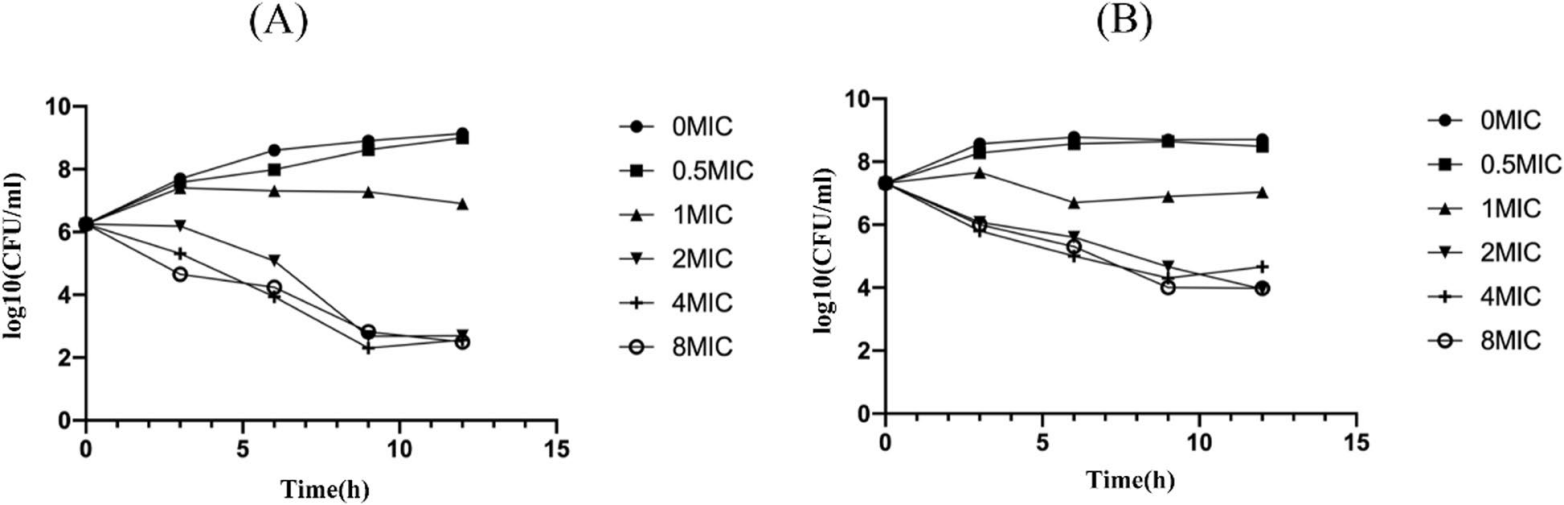
In vitro time bactericidal curve of rifaximin against E. coli ATCC 25,922 with different initial bacterial load. **A** 10^6^ CFU/ml initial inoculum group. **B** 10^7^ CFU/ml initial inoculum group

The two initial different concentrations (10^6^ and 10^7^ CFU/ml) of *E. coli* ATCC25922 were used for in vitro time-killing curves experiment. They showed that the bactericidal activity of rifaximin conformed to time-dependence rather than concentration-dependence. The time-kill curve is shown in Fig. 1.

### Pharmacokinetics

The LOD and LOQ for determining rifaximin in mammary glands were 0.2 and 0.4 µg/g, respectively. The recoveries of rifaximin in mouse mammary glands were 84.54 ± 5.31%, 84.07 ± 7.34%, and 88.96 ± 7.16% at three different spiked concentrations (10 µg/g, 100 µg/g, and 200 µg/g), respectively, and the coefficients of variation (CV) for inter-day and intra-day values were 6.21–8.73% and 3.35–13.32%. The calibration curves for rifaximin in mammary gland tissue were linear from 1 to 200 µg/g, as shown by the correlation coefficient *R *= 0.9999. The line equation of this calibration curve was Y = 24821X-9378, where y is the peak area and x is the concentration of rifaximin in µg/g. The detailed values of the recoveries, CV for inter-day and intra-day are shown in Table 2.

**Table 2:** The average recovery, intra RSD, and inter RSD of rifaximin in the mammary gland at three spiked concentrations (10, 100, and 200 µg/g)

Spiked Concentration(µg/g)	Average Recovery(%)	SD (%)	Intra RSD(%)					Inter RSD(%)
			1 d	2 d	3 d	4 d	5 d	
10	84.54	5.31	5.31	7.43	4.19	5.29	4.04	6.21
100	84.07	7.34	7.34	3.83	3.35	4.57	6.72	8.73
200	88.96	7.16	7.16	9.95	7.87	8.75	8.98	8.04

*RSD* means relative standard deviation, *SD* means standard deviation

**Fig. 2:**
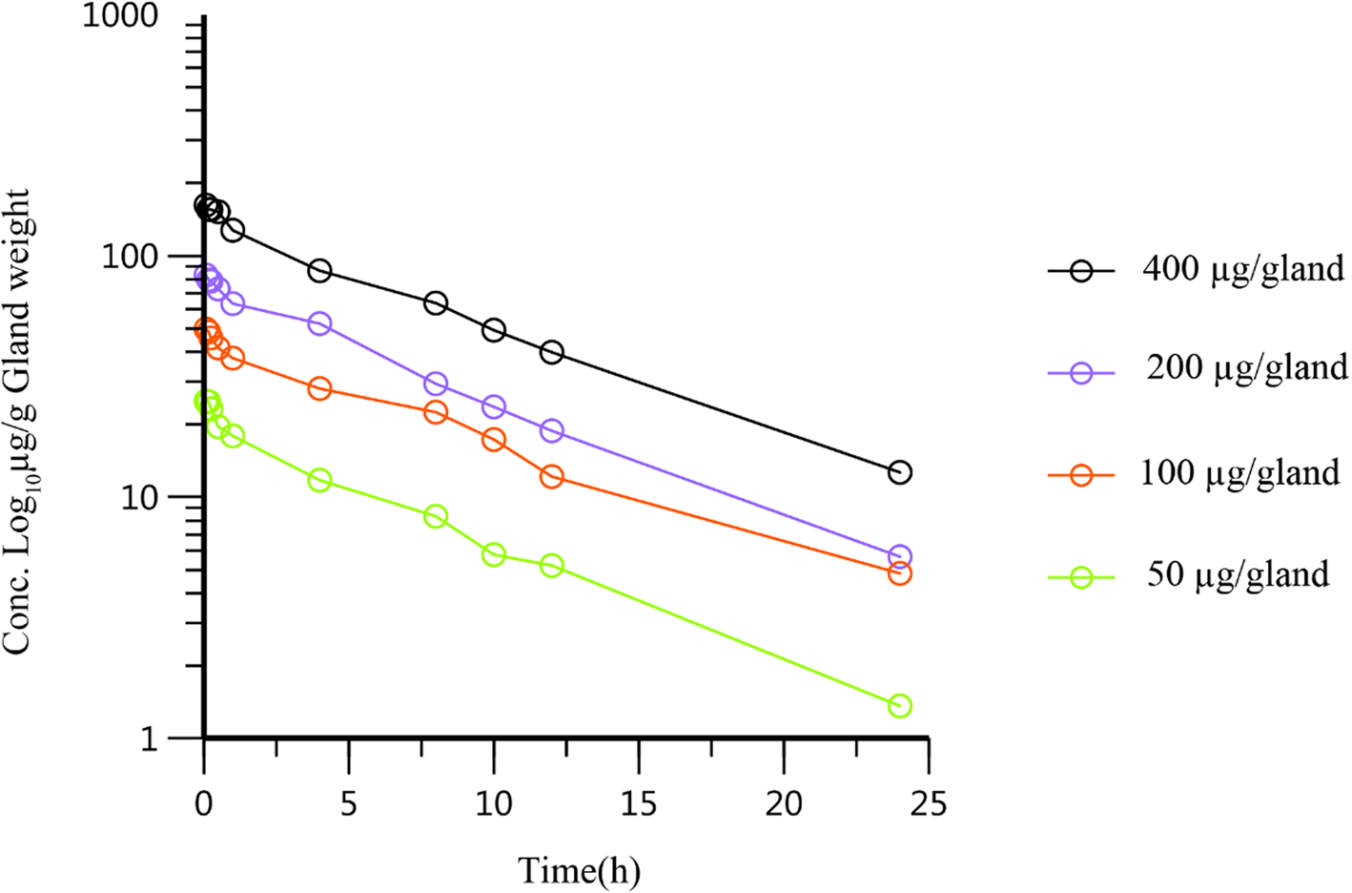
The curve of concentration vs time of rifaximin in CD-1 mouse mammary glands following intramammary administration doses of 50, 100, 200, and 400 µg/gland (*n* = 6)

The concentration of rifaximin in the mammary glands of mice in four dose groups of 50, 100, 200, and 400 µg/gland was detected by HPLC at 10 time points after administration of rifaximin. The concentration over time of rifaximin in CD-1 mouse mammary glands is shown in Fig. 2. These concentration data in the four dose groups were analyzed by the compartmental and non-compartmental model using WinNonlin software (version 8.3; Pharsight, USA). Based on the criteria of the smaller Akaike information criterion (AIC) and the better goodness of fit in the compartmental model, the two compartments model was regarded as the most suitable model of the compartmental models to analyze the concentration data. The pharmacokinetic parameters of rifaximin analyzed by the non-compartmental and the two compartment models are shown in Table 3.

**Table 3:** The main pharmacokinetic parameters of rifaximin in mammary glands following an intramammary administration with a single dose of 50, 100, 200, and 400 μg/gland in mice, analyzed by non-compartment and two-compartment models

Parameters	Administered Dose (µg/gland) (*n* = 6)				
	50	100	200	400	
non-compartment					
T_1/2_	6.18	7.45	6.84	7.19	6.92 ± 0.48
MRT	6.76	7.42	6.80	7.11	7.02 ± 0.27
AUC_24_	170.23	414.31	653.22	1287.03	
C_max_	25.01	49.73	83.17	162.17	
two-compartment models					
MRT	9.10	11.34	9.08	10.46	10.00 ± 0.96
AUC_24_	175.41	469.08	677.08	1400.29	
Cmax	27.08	53.07	87.31	165.99	
V_1_	1.85	1.88	2.29	2.41	2.11 ± 0.28
CL_1_	0.29	0.21	0.30	0.29	2.07 ± 0.04
V2	0.75	0.53	0.39	0.58	0.56 ± 0.15
CL_2_	1.13	1.21	1.14	0.40	0.97 ± 0.38

T_1/2_ is the half-life of rifaximin in the mammary gland, MRT is the average dwell time, AUC_24_ represents the area under the curve when the drug is administered for 0–24 h, C_max_ is the maximum drug concentration after drug infusion

### The effect of rifaximin against *E. coli*

**Fig. 3:**
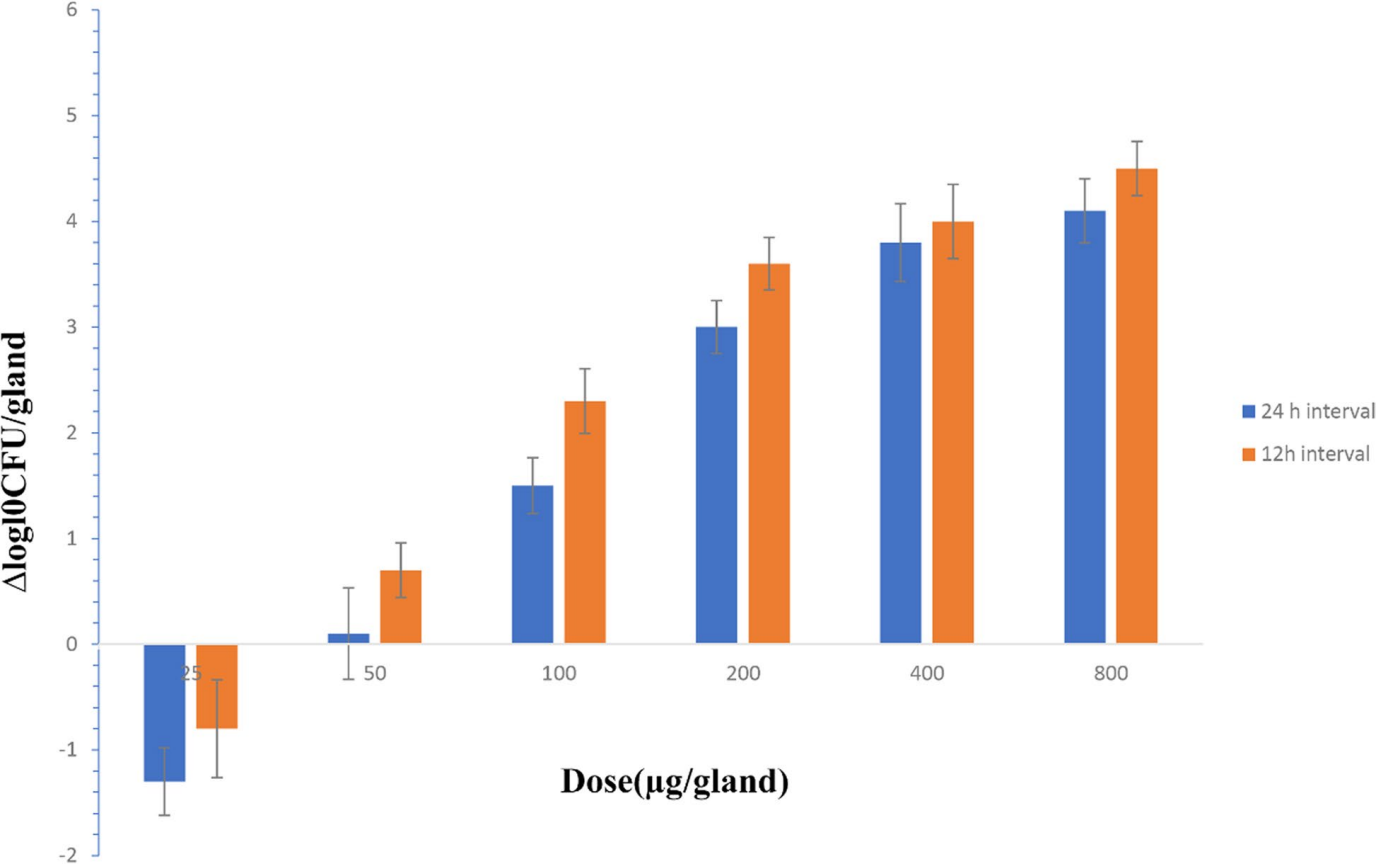
The effect of rifaximin on mastitis induced by E. coli in mouse after 12 dose regimens designed with 25, 50, 100, 200, 400, and 800 µg/gland and dosing intervals 12, and 24 h, which was expressed as ∆logl0CFU/gland

Before the infusion of rifaximin into the mammary glands of mice, the mastitis mice, induced by *E. coli*, were successfully developed and the concentration of bacteria in the mammary gland tissue reached about 8.1 log10 CFU/gland in 12 h of growth, in vivo. When the rifaximin doses of 400 µg/gland were administrated at the 12 h or 24 h interval of one day, the bactericidal effect of rifaximin demonstrated a decrease of 4 log10CFU/gland after 24 h of treatment. When 50 µg/gland of rifaximin was administered into the mammary gland in the 24-h interval of one day, it could inhibit the growth of bacteria and minimally kill bacteria in the mammary gland. The effect of rifaximin on mastitis induced by *E. coli* are presented in Fig. 3.

### PK/PD analysis

**Fig. 4:**
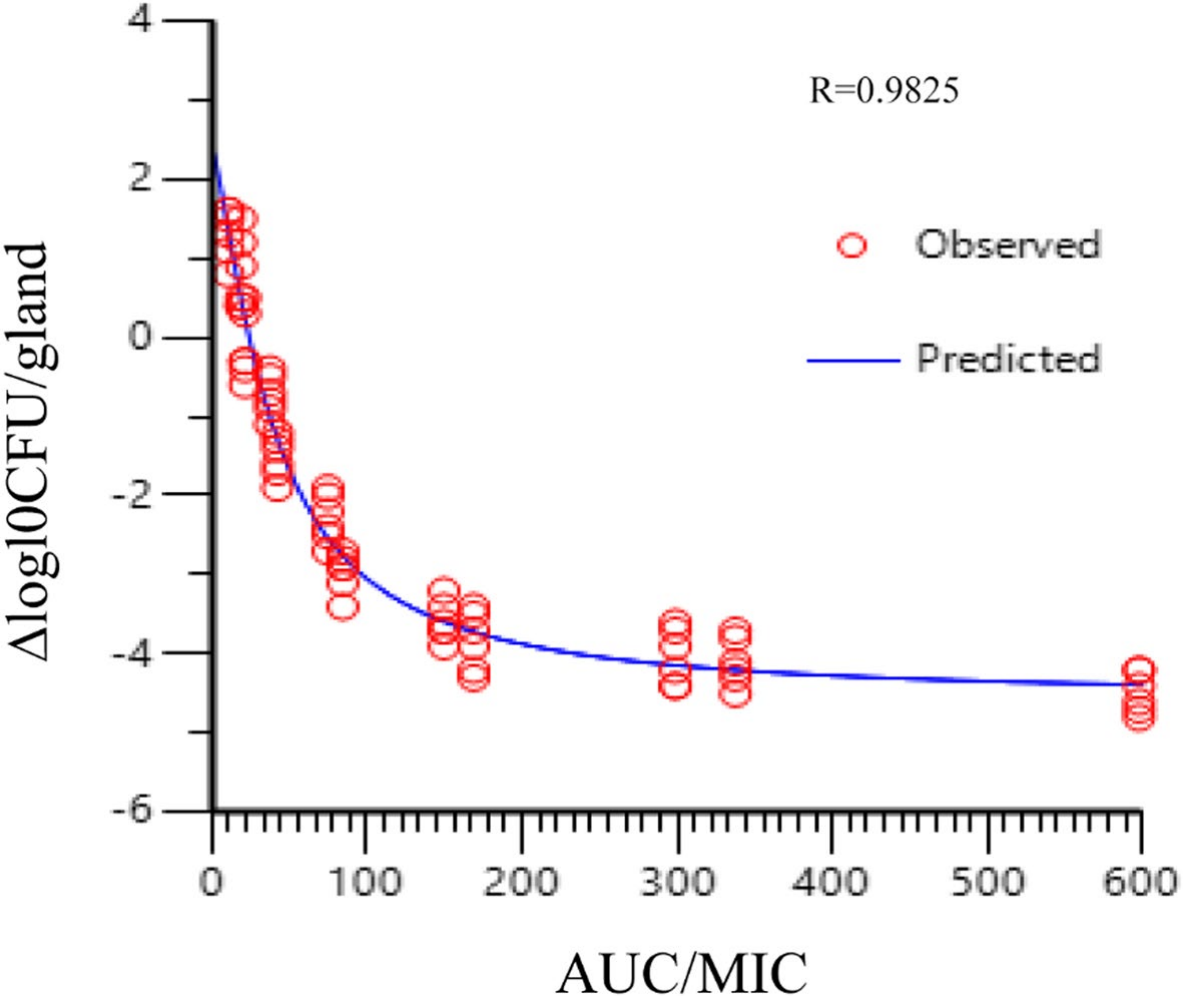
The relationship between PK/PD parameters and bactericidal effect of rifaximin (∆log10CFU/gland) analyzed by the sigmoid model. The dots represented the antibacterial effect of rifaximin (E = final log10CFU/gland-initial logl0CFU/gland) and the line represented the predicted value of E

The sigmoid E_max_ inhibitory effect model with baseline was used to fit the relationship between the pharmacokinetics of rifaximin in the mammary gland and its antibacterial effect. The surrogate AUC/MIC had high fitting degree with a correlation coefficient of 0.9825. The E_0_, E_max_, EC_50_, correlation coefficient gamma, and other AUC/MIC parameters are described in Table 4. The best fit curve is shown in Fig. 4. The 2log_10_CFU/gland and 2.5log_10_CFU/gland decreases of *E. coli* corresponded to the target parameter values of 57.80 h and 73.63 h, respectively.

**Table 4:** The important parameters in the AUC_24_/MIC using the inhibitory form *E*_max_ sigmoid model after intramammary administration

Parameters	AUC_24_/MIC
For2 log_10_ CFU/gland reduction	57.80
For2.5 log_10_ CFU/gland reduction	73.63
Log E_max_ (log10 CFU/gland)	2.36 ± 0.50
Log E_max_-log E_0_ (log_10_ CFU/gland)	6.91 ± 0.63
EC_50_ (h)	38.85 ± 4.43
Slope (N)	1.35 ± 0.18

### Monte Carlo simulation

**Fig. 5:**
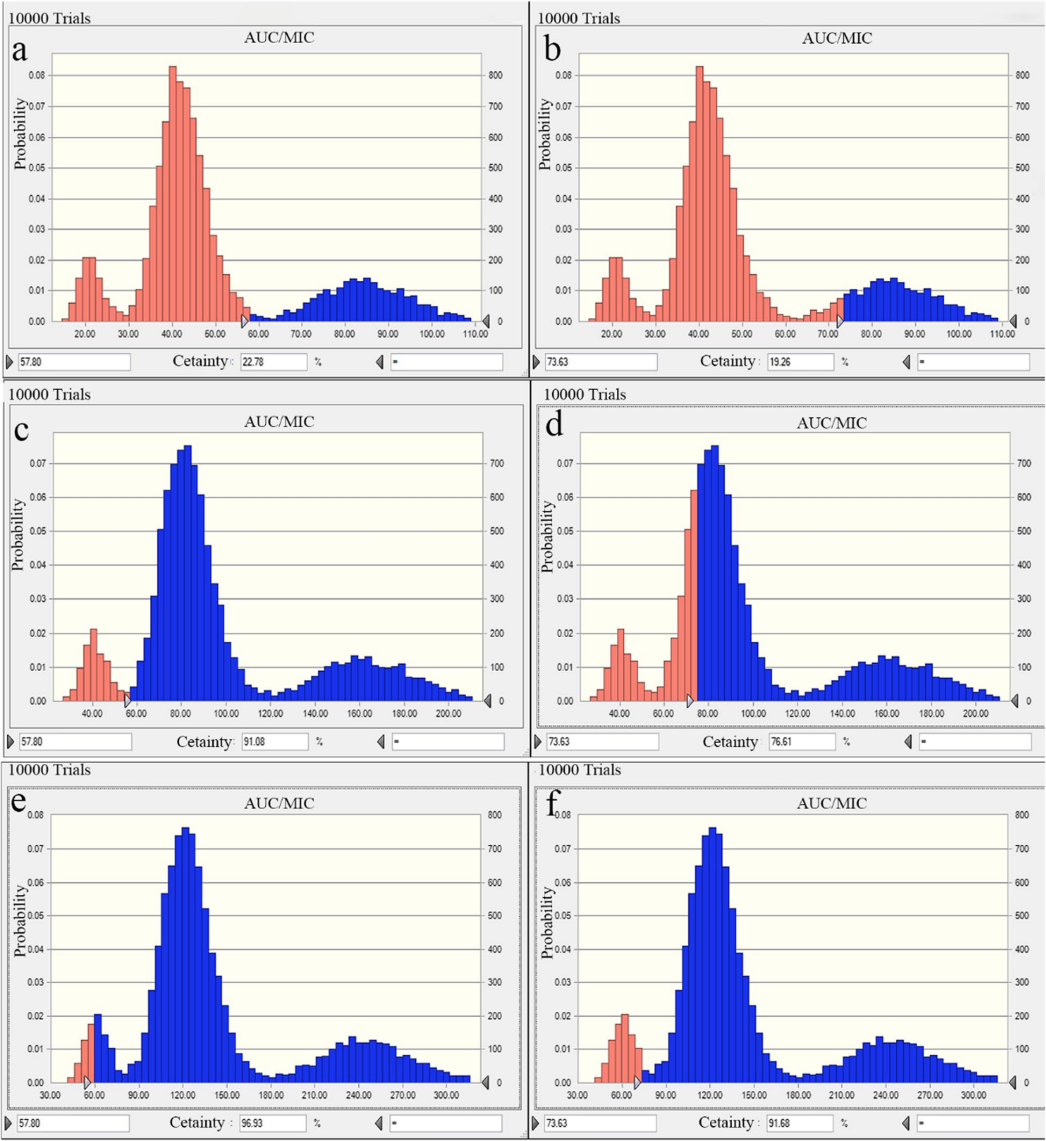
The probability distribution of AUC/MIC for rifaximin using a 10,000-subject Monte Carlo analysis based on the measured PK parameters obtained following mammary gland injection at 100 mg/gland with 24-h (**a** and **b**), 12-h (**c** and **d**) and 8-h (**e** and **f**) dosing interval in dairy cows and E. coli MIC distribution in this study. The areas of blue columns represent the probability of target attainment (PTA) for the 2log10CFU/gland decrease and the 2.5log10CFU/gland decrease of E. coli

The probability distribution of AUC/MIC parameters for 100 mg/gland rifaximin administered to the mammary gland of dairy cows once, twice, and three times a day are shown in Fig. 5. When the 2log_10_CFU/gland bacterial decline was set as the target effect value, the probability of target attainment (PTA) for two and three administrations of rifaximin in one day were all higher than 90%. If the 2.5log_10_CFU/gland bacterial reduction was also set as the target effect value, the PTA for the different three dose schemes in a day were 19.26%, 76.61%, and 91.68%, respectively.

## Discussion

In this study, the ranges of MICs of rifaximin against Escherichia coli ATCC25922 and clinical strains was found to be 4–16 µg/mL, which is consistent with previous studies [20–22]. The calculated results of MIC_50_ and MIC_90_ were 4 and 8 µg/mL, respectively, and the MIC_90_ was applied to the later PK/PD analysis integration. Based on the results of MIC values above, we conclude that rifaximin has a good bactericidal effect on *E. coli* in the clinic. The in vitro bactericidal curve showed that the bactericidal effect was similar when the concentration of rifaximin was 2xMIC or higher concentrations. When the initial concentration of *E. coli* was 10^6^ µg/ml and 10^7^ µg/ml, the decline value of bacteria was a 3.5 log10CFU/ml reduction and a 3 log10CFU/ml reduction. Therefore, rifaximin can kill more than 99.9% of *E. coli* ATCC25922 in the two different initial bacterial concentration groups. These findings are similar to Yu’s and Xiao’s report [23, 24]. However, rifaximin could not inhibit the growth of *E. coli* when the drug concentration was lower than MIC. Based on the results above, different initial concentrations of *E. coli* have an effect on the bactericidal amount of rifaximin for 12 h. Guo also obtained similar results using different initial inoculum of *E. coli*, which had an impact on the bactericidal effect of cefquinome [25]. Debbia plotted a bactericidal curve of rifaximin based on in vitro data using 10^6^–10^7^ CFU/ml and 10^7^–10^8^ CFU/ml of initial inoculum of *E. coli*. The results showed that the bacterial decline values of 10^6^–10^7^ CFU/ml and 10^7^–10^8^ CFU/ml of initial inoculum groups were almost 4 log10 CFU/ml with 2xMIC or higher concentrations of rifaximin [21]. These results are similar to those of our study and demonstrate that the bactericidal activity of rifaximin is time-dependent, and not concentration-dependent. Based on the in vivo pharmacodynamics of rifaximin on mouse mastitis induced by *E. coli* ATCC25922, rifaximin could not kill the bacteria in the mammary gland tissue of mice when it was given as one or two doses of 25 µg/gland in a day. When the dosage was increased to more than 50 µg/gland 1 times in a day, rifaximin can kill the bacteria in the gland tissue of mice. Additionally, when the dosage was 200 µg/gland or above, the bactericidal effect of rifaximin changed little with the increased dosage. The maximum therapeutic effective of rifaximin is 4 log10 CFU/gland decreases when the dose is 400 µg/gland for 12 h a day. Yu studied the pharmacodynamics of cefquinome on mastitis mice, induced by *E. coli*, and the maximum effective of cefquinome was 6 log10 CFU/gland decreases in a 24-h therapeutic treatment with a dose of 400 µg/gland once a day [16].

In our previous study, we administered rifaximin to one mammary gland of the fourth pair of mice, and the results showed that the drug concentration was not detected or close to the detection limit in the untreated mammary gland on opposite side [17]. Histologically, the close link between secretory cells at their apex by tight junctions in the lactating udder forms the blood–milk barrier, which accounts for the passive transport of drugs between the blood and milk [26]. The fourth gland tissue of mice are relatively large, clear in structure, and easy to obtain [14]. Therefore, we selected the fourth pair of the mammary gland for the pharmacokinetic experiment in mice. In the pharmacokinetics study of the mammary gland, we choose a two-compartment model based on the criteria of the smaller Akaike information criterion (AIC) and non-compartment model. The results showed the elimination half-life (t_1/2_) and mean retention time (MRT) were 5.46 h and 10.0 h for the two-compartment model, respectively, and 6.92 h and 7.02 h for non-compartment model, respectively. These findings indicate that rifaximin can reach a high concentration and remain in the mouse mammary gland for a long time.

In the pharmacodynamic experiments of rifaximin on mouse mastitis, we injected 50 µL of *E. coli* bacterial fluid (containing 10^6^ CFU/gland) into the mammary gland of mice under an anatomical microscope through a micro syringe. After 12 h of growth, the bacterial concentration in the mammary gland reached 10^8^ CFU/gland. During this period, the activity, food intake, and water consumption of mice decreased. Both sides of the abdomen of the fourth pair of mammary glands were concave and rigid, with a slight red hue observed in the abdomen. After dissection, the mammary glands of mice were swollen, slightly red, and had a foul smell. These results are consistent with earlier studies [17, 27, 28], indicating that mouse mastitis induced by *E. coli* was successfully achieved.

In our previous study, we found that the suitable parameter of PK/PD model is AUC/MIC for rifaximin. In this experiment, we demonstrated that PK/PD surrogate AUC/MIC had a good linear relationship with the antibacterial effect of rifaximin with *R* = 0.9825. So, the PK/PD surrogate AUC/MIC was used to calculate the target value for the different antibacterial effects. In the inhibitory E_max_ sigmoid model, the E_max_ (log_10_ CFU/gland), E_max_ − E_0_ (log_10_ CFU/gland), EC_50_, and Slope (N) were 2.36 ± 0.50, 6.91 ± 0.63, 2.36 ± 0.50, and 1.35 ± 0.18, respectively. When the target value was set to 2logCFU/gland and 2.5logCFU/gland decreases, the corresponding values of AUC/MIC were 57.80 and 73.63 h.

In the Monte Carlo simulation, the PTAs of rifaximin were 22.78%, 91.08%, and 96.93% for 2logCFU/gland, and 19.26%, 76.61%, and 91.68% for 2.5logCFU/gland in 100 mg/gland administered once, twice, and three times in 24 h, respectively. The PTAs of rifaximin were all higher than 75% when the dose schemes were 100 mg/gland, administered twice and three times in 24 h. Clinically, given the high profits, the scheme of 100 mg/gland administered twice times in 24 h was selected. Based on the results of the Monte Carlo simulation, we concluded that the administration scheme for rifaximin was reasonable in curing mastitis in cows, and rifaximin has a significant antibacterial effect. Compared to Wang’s report [17], when the bacterial decline value was set to 2logCFU/gland decreases, the PTA of rifaximin in the treatment of *E. coli* was greater than that of *S. aureus* for the administered once, twice, and three times in the 24-h groups. If dairy cows are diagnosed with mastitis caused by *S. aureus* and *E. coli* infection simultaneously, mastitis cows induced by *S. aureus* should be given priority in the treatment process as the dosage required for the treatment of *S. aureus* is more than that for the treatment of *E. coli* mastitis. Due to differences in species between cows and mice, and the gap between the mouse mastitis model and the cow mastitis model, we cannot directly translate the results of this study to mastitis cows. However, the colonization of bacteria in the mammary glands of mice is similar to the colonization of bacteria in the mammary glands of dairy cows. The mouse mastitis model provides a milky and representative growth environment of serum, allowing pathogens to interact with host mammary cells and immune components during infection [14]. Therefore, cow and mouse infection models have relatively similar PK/PD indices. Additionally, the huge cost of in vivo PK/PD modeling in lactation cows inhibits the ability to directly study this in cows. Taken together, the in vivo PK/PD integration of rifaximin in mice against *E. coli* provides fundamental data and rational for the use of rifaximin in bovine mastitis therapy.

## Conclusion

In summary, this is the first study that assesses mouse mammary gland tissue PK/PD integration for investigating the effectiveness of rifaximin for curing mastitis induced by *E. coli*. The clinical effect of rifaximin on dairy cow mastitis was evaluated by the Monte Carlo model. The clinically recommended dosage regimen of 100 mg/gland every 12 h in one day achieved a 91.08% and 76.61% cure rate for the decrease of 2log10CFU/gland and 2.5log10CFU/gland in the treatment of bovine mastitis caused by *Escherichia coli* infection.

## Supplementary Information

**Additional file 1:** **Supplementary Table 1.** The antibacterial effect of rifaximin (E = final log10CFU/gland – initial logl0CFU/gland). **Supplementary Table 2.** In vitro time bactericidal curve of rifaximin against *E. coli* ATCC 25922 with 10^6^ CFU/ml initial bacterial load. **Supplementary Table 3.** In vitro time bactericidal curve of rifaximin against *E. coli* ATCC 25922 with 10^7^ CFU/ml initial bacterial load. **Supplementary Table 4.** The concentration of rifaximin in CD-1 mouse mammary glands following intramammary administration doses of 400 ug/gland. (ug/g). **Supplementary Table 5.** The concentration of rifaximin in CD-1 mouse mammary glands following intramammary administration doses of 200 ug/gland. (ug/g). **Supplementary Table 6.** The concentration of rifaximin in CD-1 mouse mammary glands following intramammary administration doses of 100 ug/gland. (ug/g). **Supplementary Table 7.** The concentration of rifaximin in CD-1 mouse mammary glands following intramammary administration doses of 50 ug/gland. (ug/g). **Supplementary Table 8.** The relationship between PK/PD parameters and bactericidal effect of rifaximin (∆log10CFU/gland).

## Data Availability

All data generated or analyzed during this study are included in this published article and its supplementary information files.
