# Supplementary material for: A PK/PD model for the evaluation of clinical rifaximin dosage for the treatment of dairy cow mastitis induced by Escherichia coli
Source: BMC Vet Res. 2023 Jan 21;19:19. doi: 10.1186/s12917-022-03564-2 (PMC9863146; doi:10.1186/s12917-022-03564-2)
Supplement: Supplementary file 1 — Additional file 1: Supplementary Table 1. The antibacterial effect of rifaximin (E = final log10CFU/gland – initial logl0CFU/gland). Supplementary Table 2. In vitro time bactericidal curve of rifaximin against E. coli ATCC 25922 with 106 CFU/ml initial bacterial load. Supplementary Table 3. In vitro time bactericidal curve of rifaximin against E. coli ATCC 25922 with 107 CFU/ml initial bacterial load. Supplementary Table 4. The concentration of rifaximin in CD-1 mouse mammary glands following intramammary administration doses of 400 ug/gland. (ug/g). Supplementary Table 5. The concentration of rifaximin in CD-1 mouse mammary glands following intramammary administration doses of 200 ug/gland. (ug/g). Supplementary Table 6. The concentration of rifaximin in CD-1 mouse mammary glands following intramammary administration doses of 100 ug/gland. (ug/g). Supplementary Table 7. The concentration of rifaximin in CD-1 mouse mammary glands following intramammary administration doses of 50 ug/gland. (ug/g). Supplementary Table 8. The relationship between PK/PD parameters and bactericidal effect of rifaximin (∆log10CFU/gland). [file 12917_2022_3564_MOESM1_ESM.docx]

**NOTE: The following are the raw data of this experiment.**

Supplementary Table1. The antibacterial effect of rifaximin (E = final log10CFU/gland – initial logl0CFU/gland)

| Dose（µg/gland） | Administration interval | Administration interval |
| --- | --- | --- |
|  | 24h | 12h |
| 25 | -1.5 | -0.4 |
| 25 | -0.8 | -1.5 |
| 25 | -1.6 | -1.2 |
| 25 | -1.3 | -0.4 |
| 25 | -1.1 | -0.5 |
| 25 | -1.6 | -0.9 |
| Average | -1.3166667 | -0.8166667 |
| SD | 0.31885211 | 0.46224092 |
| 50 | -0.3 | 0.7 |
| 50 | -0.5 | 0.9 |
| 50 | 0.3 | 0.5 |
| 50 | 0.6 | 1.1 |
| 50 | 0.3 | 0.8 |
| 50 | 0.4 | 0.4 |
| Average | 0.13333333 | 0.73333333 |
| SD | 0.43204938 | 0.25819889 |
| 100 | 1.6 | 2.2 |
| 100 | 1.3 | 2 |
| 100 | 1.4 | 2.5 |
| 100 | 1.2 | 2.7 |
| 100 | 1.7 | 2.4 |
| 100 | 1.9 | 1.9 |
| Average | 1.51666667 | 2.28333333 |
| SD | 0.26394444 | 0.3060501 |
| 200 | 2.7 | 3.6 |
| 200 | 2.9 | 3.7 |
| 200 | 3.1 | 3.2 |
| 200 | 3.4 | 3.4 |
| 200 | 2.8 | 3.9 |
| 200 | 2.9 | 3.7 |
| Average | 2.96666667 | 3.58333333 |
| SD | 0.25033311 | 0.24832774 |
| 400 | 3.9 | 4.4 |
| 400 | 4.3 | 4.2 |
| 400 | 4.2 | 4.4 |
| 400 | 3.7 | 3.7 |
| 400 | 3.4 | 3.6 |
| 400 | 3.5 | 3.9 |
| Average | 3.83333333 | 4.03333333 |
| SD | 0.36696957 | 0.35023801 |
| 800 | 4.3 | 4.6 |
| 800 | 4.5 | 4.4 |
| 800 | 4.2 | 4.8 |
| 800 | 4.1 | 4.2 |
| 800 | 3.7 | 4.2 |
| 800 | 3.8 | 4.7 |
| Average | 4.1 | 4.48333333 |
| SD | 0.30331502 | 0.25625508 |

Supplementary Table2. In vitro time bactericidal curve of rifaximin against *E. coli* ATCC 25922 with 10^6^ CFU/ml initial bacterial load.

| time | rifaximin concentrations | | | | | |
| --- | --- | --- | --- | --- | --- | --- |
|  | control | 0.5MIC | 1MIC | 2MIC | 4MIC | 8MIC |
| 0 | 6.255273 | 6.255273 | 6.255273 | 6.2552725 | 6.255273 | 6.25527 |
| 3h | 7.69897 | 7.580363 | 7.40515 | 6.184691 | 5.30963 | 4.653213 |
| 6h | 8.60309 | 7.985098 | 7.313943 | 5.077121 | 3.934498 | 4.234026 |
| 9h | 8.90618 | 8.62 | 7.277121 | 2.677121 | 2.301703 | 2.823746 |
| 12h | 9.133539 | 8.995635 | 6.90309 | 2.69897 | 2.55897 | 2.497 |

Supplementary Table3. In vitro time bactericidal curve of rifaximin against *E. coli* ATCC 25922 with 10^7^ CFU/ml initial bacterial load.

| time | rifaximin concentrations | | | | | |
| --- | --- | --- | --- | --- | --- | --- |
|  | control | 0.5MIC | 1MIC | 2MIC | 4MIC | 8MIC |
| 0 | 7.322183 | 7.322183 | 7.322183 | 7.322183 | 7.322183 | 7.322183 |
| 3h | 8.568202 | 8.278754 | 7.662758 | 6.079181 | 5.80103 | 6 |
| 6h | 8.778151 | 8.568202 | 6.70309 | 5.60206 | 5 | 5.30103 |
| 9h | 8.69897 | 8.644068 | 6.89897 | 4.662758 | 4.3 | 4.00309 |
| 12h | 8.70206 | 8.491362 | 7.033424 | 3.954243 | 4.662758 | 3.98227 |

| time（hr） |  |  |  |  |  |  | AVG |
| --- | --- | --- | --- | --- | --- | --- | --- |
| 0.08 | 186.05 | 143.58 | 162.12 | 150.62 | 159.10 | 173.15 | 162.44 |
| 0.17 | 163.21 | 152.17 | 147.36 | 153.62 | 156.23 | 162.42 | 155.84 |
| 0.25 | 158.27 | 146.17 | 162.43 | 154.35 | 163.17 | 148.30 | 155.45 |
| 0.5 | 148.06 | 154.02 | 143.26 | 138.51 | 176.53 | 154.32 | 152.45 |
| 1 | 132.12 | 126.34 | 118.22 | 135.76 | 146.16 | 108.20 | 127.80 |
| 4 | 96.53 | 102.89 | 87.26 | 99.42 | 67.26 | 69.16 | 87.09 |
| 8 | 63.84 | 68.23 | 56.33 | 73.26 | 58.08 | 64.62 | 64.06 |
| 10 | 57.69 | 48.02 | 53.13 | 47.34 | 47.36 | 43.10 | 49.44 |
| 12 | 41.22 | 37.65 | 51.19 | 42.86 | 36.92 | 32.26 | 40.35 |
| 24 | 15.12 | 9.68 | 13.53 | 17.29 | 10.35 | 12.11 | 13.01 |

Supplementary Table4. The concentration of rifaximin in CD-1 mouse mammary glands following intramammary administration doses of 400 ug/gland. (ug/g)

Supplementary Table5. The concentration of rifaximin in CD-1 mouse mammary glands following intramammary administration doses of 200 ug/gland. (ug/g)

| time（hr） |  |  |  |  |  |  | AVG |
| --- | --- | --- | --- | --- | --- | --- | --- |
| 0.08 | 79.85 | 88.32 | 102.14 | 68.31 | 79.30 | 83.62 | 83.59 |
| 0.17 | 82.21 | 89.06 | 78.56 | 73.62 | 75.30 | 80.18 | 79.82 |
| 0.25 | 76.12 | 78.30 | 82.15 | 81.42 | 82.19 | 72.35 | 78.76 |
| 0.5 | 79.12 | 76.86 | 75.25 | 69.33 | 71.26 | 67.12 | 73.16 |
| 1 | 58.48 | 61.12 | 70.47 | 56.10 | 72.62 | 63.21 | 63.67 |
| 4 | 55.03 | 58.46 | 51.78 | 49.26 | 58.12 | 43.26 | 52.65 |
| 8 | 32.26 | 23.03 | 25.84 | 29.56 | 27.29 | 41.78 | 29.96 |
| 10 | 21.08 | 28.55 | 34.15 | 19.16 | 16.82 | 24.26 | 24.00 |
| 12 | 18.26 | 14.78 | 21.26 | 23.30 | 19.59 | 18.42 | 19.27 |
| 24 | 5.89 | 7.21 | 5.26 | 3.58 | 6.08 | 8.53 | 6.09 |

Supplementary Table6. The concentration of rifaximin in CD-1 mouse mammary glands following intramammary administration doses of 100 ug/gland. (ug/g)

| time（hr） |  |  |  |  |  |  | AVG |
| --- | --- | --- | --- | --- | --- | --- | --- |
| 0.08 | 50.47 | 40.62 | 55.49 | 61.69 | 48.36 | 43.19 | 49.73 |
| 0.17 | 47.12 | 45.62 | 56.13 | 57.05 | 39.26 | 46.06 | 48.33 |
| 0.25 | 51.21 | 41.84 | 48.72 | 43.09 | 49.76 | 42.81 | 45.67 |
| 0.5 | 43.03 | 37.01 | 42.81 | 47.92 | 41.36 | 39.04 | 41.50 |
| 1 | 40.20 | 34.72 | 40.29 | 34.61 | 38.53 | 40.59 | 37.67 |
| 4 | 30.23 | 34.19 | 27.75 | 26.23 | 24.29 | 28.06 | 28.17 |
| 8 | 24.52 | 26.03 | 19.19 | 21.26 | 28.08 | 17.63 | 22.50 |
| 10 | 17.89 | 13.39 | 18.21 | 19.03 | 21.01 | 16.10 | 17.33 |
| 12 | 11.20 | 9.19 | 14.13 | 16.52 | 14.26 | 9.14 | 12.17 |
| 24 | 5.62 | 6.12 | 5.86 | 6.13 | 3.14 | 4.30 | 4.83 |

Supplementary Table7. The concentration of rifaximin in CD-1 mouse mammary glands following intramammary administration doses of 50 ug/gland. (ug/g)

| time（hr） |  |  |  |  |  |  | AVG |
| --- | --- | --- | --- | --- | --- | --- | --- |
| 0.08 | 26.47 | 27.12 | 26.62 | 28.24 | 22.15 | 19.48 | 25.01 |
| 0.17 | 28.43 | 24.07 | 19.52 | 26.14 | 29.25 | 21.62 | 24.84 |
| 0.25 | 24.06 | 22.13 | 19.83 | 25.26 | 25.3 | 22.8 | 23.23 |
| 0.5 | 18.25 | 24.03 | 18.25 | 17.43 | 23.06 | 16.54 | 19.59 |
| 1 | 17.62 | 15.62 | 18.62 | 21.05 | 16.53 | 18.25 | 17.95 |
| 4 | 10.23 | 13.62 | 15.29 | 11.05 | 10.85 | 9.52 | 11.76 |
| 8 | 9.62 | 6.25 | 10.62 | 7.35 | 8.52 | 7.65 | 8.34 |
| 10 | 5.32 | 7.32 | 4.29 | 3.85 | 6.51 | 7.39 | 5.78 |
| 12 | 6.95 | 5.32 | 4.85 | 6.23 | 4.1 | 3.82 | 5.21 |
| 24 | 1.13 | 1.26 | 1.2 | 2.01 | 1.46 | 1.13 | 1.37 |

Supplementary Table8. The relationship between PK/PD parameters and bactericidal effect of rifaximin (∆log10CFU/gland).

| **AUC/MIC** | △log CFU/gland | **AUC/MIC** | △log CFU/gland | **AUC/MIC** | △log CFU/gland |
| --- | --- | --- | --- | --- | --- |
| 74.67978875 | 2.2 | 10.5394855 | -1.5 | 597.4383125 | 4.6 |
| 74.67978875 | 2 | 10.5394855 | -0.8 | 597.4383125 | 4.4 |
| 74.67978875 | 2.5 | 10.5394855 | -1.6 | 597.4383125 | 4.8 |
| 74.67978875 | 2.7 | 10.5394855 | -1.3 | 597.4383125 | 4.2 |
| 74.67978875 | 2.4 | 10.5394855 | -1.1 | 597.4383125 | 4.2 |
| 74.67978875 | 1.9 | 10.5394855 | -1.6 | 597.4383125 | 4.7 |
| 42.1579425 | 1.6 | 298.71915 | 4.4 | 337.2635375 | 4.3 |
| 42.1579425 | 1.3 | 298.71915 | 4.2 | 337.2635375 | 4.5 |
| 42.1579425 | 1.4 | 298.71915 | 4.4 | 337.2635375 | 4.2 |
| 42.1579425 | 1.2 | 298.71915 | 3.7 | 337.2635375 | 4.1 |
| 42.1579425 | 1.7 | 298.71915 | 3.6 | 337.2635375 | 3.7 |
| 42.1579425 | 1.9 | 298.71915 | 3.9 | 337.2635375 | 3.8 |
| 149.359575 | 3.6 | 168.631775 | 3.9 |  |  |
| 149.359575 | 3.7 | 168.631775 | 4.3 |  |  |
| 149.359575 | 3.2 | 168.631775 | 4.2 |  |  |
| 149.359575 | 3.4 | 168.631775 | 3.7 |  |  |
| 149.359575 | 3.9 | 168.631775 | 3.4 |  |  |
| 149.359575 | 3.7 | 168.631775 | 3.5 |  |  |
| 84.315885 | 2.7 | 37.339895 | 0.7 |  |  |
| 84.315885 | 2.9 | 37.339895 | 0.9 |  |  |
| 84.315885 | 3.1 | 37.339895 | 0.5 |  |  |
| 84.315885 | 3.4 | 37.339895 | 1.1 |  |  |
| 84.315885 | 2.8 | 37.339895 | 0.8 |  |  |
| 84.315885 | 2.9 | 37.339895 | 0.4 |  |  |
| 18.6699475 | -0.4 | 21.07897125 | -0.3 |  |  |
| 18.6699475 | -1.5 | 21.07897125 | -0.5 |  |  |
| 18.6699475 | -1.2 | 21.07897125 | 0.3 |  |  |
| 18.6699475 | -0.4 | 21.07897125 | 0.6 |  |  |
| 18.6699475 | -0.5 | 21.07897125 | 0.3 |  |  |
| 18.6699475 | -0.9 | 21.07897125 | 0.4 |  |  |
